# Effects of Multileaf Collimator Design and Function When Using an Optimized Dynamic Conformal Arc Approach for Stereotactic Radiosurgery Treatment of Multiple Brain Metastases With a Single Isocenter: A Planning Study

**DOI:** 10.7759/cureus.9833

**Published:** 2020-08-18

**Authors:** Michael Taylor, Jamone Williams, John F Gleason

**Affiliations:** 1 Medical Physics, Alliance Cancer Care, Huntsville, USA; 2 Radiation Oncology, Alliance Cancer Care, Huntsville, USA

**Keywords:** srs, fsrs, brain metastases, radiosurgery, stereotactic radiosurgery, vmat, dynamic conformal arc, single isocenter, multi-isocenter, fractionated srs

## Abstract

Background

Stereotactic radiosurgery (SRS) or fractionated SRS (fSRS) are effective options for the treatment of brain metastases. When treating multiple metastases with a linear accelerator-based approach, a single isocenter allows for efficient treatment delivery. In this study, we present our findings comparing dosimetric parameters of Brainlab (Munich, Germany) Elements™ Multiple Brain Mets SRS (MME) software (version 1.5 versus version 2.0) for a variety of scenarios and patients. The impact of multileaf collimator design and function on plan quality within the software was also evaluated.

Materials and methods

Twenty previously treated patients with a total of 58 lesions (from one to seven lesions each) were replanned with an updated version of the multiple brain Mets software solution. For each plan, the mean conformity index (CI), mean gradient index (GI), the volume of normal brain receiving 12 Gy (V_12_), and mean brain dose were evaluated. Additionally, all v2.0 plans were further evaluated with jaw tracking for by Elekta (Stockholm, Sweden) and HD120™ multileaf collimator by Varian Medical Systems (Palo Alto, USA).

Results

The new software version demonstrated improvements for CI, GI and V_12_ (p <0.01). For the Elekta Agility™ multileaf collimator, jaw tracking improved all dosimetric parameters except for CI (p =0.178) and mean brain dose (p =0.93). For the Varian with HD120 multileaf collimator, all parameters improved.

Conclusions

The software enhancements in v2.0 of the software provided improvements in planning efficiency and dosimetric parameters. Differences in multileaf collimator design may provide an additional incremental benefit in a subset of clinical scenarios.

## Introduction

Brain metastases (BM) are frequent sequelae of cancer estimated to occur in 9- 17% of patients with lung cancer, breast cancer, and melanoma and are the most frequent to develop brain metastases [1]. With improved detection, surveillance, and systemic therapies, the number of patients diagnosed with BM is expected to increase [2]. At our institution, the previous primary radiotherapy option for those patients with multiple brain metastases (MBM), notably more than three metastases, had been whole-brain radiotherapy (WBRT). Unfortunately, WBRT is associated with increased neurocognitive toxicity, particularly in patients expected to live longer than six months. Using a stereotactic radiosurgery (SRS) approach provides better preservation of neurocognitive function [3]. The shorter treatment course with SRS, as compared to WBRT, has the added advantages of convenience for the patient and fewer delays in systemic therapy.

The traditional SRS approach for single lesions is to treat with a single fraction of 15 - 24 Gy delivered with a margin appropriate circular collimator. Gamma Knife® delivery has been a popular option due to the accuracy of associated frame placement and target localization [4]. For larger target volumes (singular or multiple cumulative) and those adjacent to the organ at risk volumes (OAR), fractionated SRS (fSRS) in two to five fractions provides a compelling alternative with reduced toxicity compared to single-fraction SRS [5, 6]. Recent literature has demonstrated that a linear accelerator (linac), multileaf collimator (MLC) approach with appropriate software optimization and machine delivery can provide a dosimetric improvement over circular cone delivery, particularly for irregularly shaped targets and those near OARs [7].

Image-guided radiation therapy (IGRT) and frameless patient immobilization have made linac based SRS and fSRS a practical and favorable treatment option as IGRT can improve delivery accuracy and allow for appropriate gross tumor volume (GTV) to planning target volume (PTV) margin selection. Recent literature suggests that a 1.0 mm GTV expansion is appropriate and yields a lower risk of radionecrosis than a 3.0 mm margin [8].

When performing linac based SRS or fSRS for MBM, one of the traditional challenges is that each of the treated BM requires a unique isocenter. Depending on the MBM relative spatial distribution and proximity to OARs, planning can become tedious and time-consuming. Additionally, multiple isocenter and multiple target plans require significant time and department resources to verify and prolonged treatment time to deliver. Several investigations have demonstrated that a volumetric modulated arc therapy (VMAT) approach provides an opportunity to overcome dosimetric challenges as well as to deliver with a single isocenter, thus reducing treatment delivery time [9]. Due to the nature and complexity of MBM treatment with VMAT, plan optimization and quality assurance can also be time and resource consuming.

Elements™ Multiple Brain Mets SRS (MME) is a template-based, optimized dynamic conformal arc planning tool that can treat one or more targets simultaneously. Predefined templates include a couch, allowable collimator, and gantry angles. Once targets are defined, the isocenter is placed at the geometric center of all treatment targets. At each couch angle, one or more dynamic conformal arcs can be delivered according to the template selected [10].

The primary goal of this analysis is to compare the dosimetric parameters of MME version 1.5 (v1.5) with the newly released MME version 2.0 (v2.0) on an Elekta VersaHD™ (Elekta AB, Stockholm, Sweden) equipped with an Agility™ 160 leaf MLC, with and without jaw tracking. The jaw tracking feature on the Elekta Agility MLC allows the jaw to block with a higher resolution in the direction of jaw travel, which can improve the conformity of peripheral targets during a given arc pass. It’s important to note that peripheral targets might vary between arcs depending on resultant optimization parameters. Additionally, a Varian HD120™ MLC was evaluated and included in the analysis.

MME v2.0 provides additional features not present in v1.5 that improve plan quality. The addition of gradient index (GI) optimization parameters can improve dose falloff around a given target. This is accomplished by narrowing the target margin, which will increase both target inhomogeneity and maximum dose and, depending on target shape, improve prescription dose conformity. Another new feature, OAR sparing, allows the user to improve on dose constraints when critical structures are close to targets. Essentially, OARs can be identified during optimization and partially shielded by the MLC during a given arc pass resulting in decreased dose to a given OAR.

## Materials and methods

Equipment

MME v1.5 was commissioned on an Elekta VersaHD linac with two high-dose-rate flattening filter-free (FFF) and two flattened photon modes. The models were validated, and a comprehensive quality assurance process was established according to the recommendations of the American Association of Physicists in Medicine Practice Guidelines 5.a, 8.a, and 9.a [11-13]. All cranial SRS and fSRS treatments are performed with the 6 FFF mode, and this was the only energy evaluated in this planning study. MME v1.5 experience has previously been presented [14].

The Elekta VersaHD comes equipped with the Agility MLC (Elekta AB, Stockholm, Sweden), which consists of 160 interdigitating leaves, projecting to a 5.0 mm effective width at the isocenter. The individual leaves can travel at approximately 3.5 cms^−1, ^and the leaf guides can travel at approximately 3.0 cms^−1^. Leaf transmission is less than 0.4% across all energies. Both the leaves and the X-jaws can modulate in 1.0 mm increments.

The Varian HD120 is not in current use at our facility, so a vendor-supplied, pre-configured beam model was used (Varian Medical Systems, Palo Alto, USA) for plan comparisons. This model consisted of a TrueBeam STx with a 6 FFF mode, which was used to recreate all SRS and fSRS treatments. The Varian HD120 MLC consists of 120 interdigitating leaves and a 1.0 mm positional resolution. The central 8 cm of leaves are 2.5 mm wide, and the outboard 14 cm leaves are 5.0 mm wide. The maximum leaf speed is approximately 2.5 cms^−1^. Leaf transmission is less than 1.4% [15].

Patient population and treatment criteria

For this study, twenty v1.5 MME patient plans were selected that had been previously treated at our institution. These patients represent a broad range in the number and size of targets representative of our patient population. High-resolution contrast-enhanced MRI images, along with corresponding CT images, are obtained and sent to the MME treatment planning system. The GTV is delineated on the MRI and fused with CT. The patient characteristics for this study are shown in Table 1. For each patient, the number of lesions, the prescription dose per lesion, the number of fractions, and the cumulative PTV volume of all lesions, as well as the minimum and maximum volume of each patient’s lesions are presented. Brainlab MME v1.5 requires all plans to be generated from a pre-configured template. These templates call for three to six couch positions with one to two arcs each. Most plans consisted of four couch positions with a single arc pass. Templates with additional arcs were used when striving to improve target coverage or reduce OAR doses. Depending on total BM or MBM size, volume, and location, SRS or fSRS is selected according to the criteria outlined in Table 2. In order to evaluate the improvements in v2.0, best case plans were generated to compare to clinically treated v1.5 plans.

**Table 1:** Patients’ number of lesions, prescription dose per lesion, number of fractions per lesion, PTV sum volume, smallest and largest PTV volume characteristics PTV - planning target volume

Patient	Number of lesions	Prescription dose (Gy)	Number of fractions	PTV sum (cm^3^)	PTV MIN (cm^3^)	PTV MAX (cm^3^)
1	4	25/27.5/30/30	5	1.77	0.25	0.88
2	4	30/30/30/30	5	1.83	0.33	0.67
3	5	27/22.5/22.5/24/27	3	5.05	2.76	0.95
4	7	27/24/27/27/24/27/27	3	7.68	0.66	1.70
5	2	22/18	1	3.53	0.17	3.36
6	2	22/22	1	1.55	0.28	1.27
7	1	22	1	0.51	0.51	0.51
8	1	18	1	4.17	4.17	4.17
9	3	22/22/22	1	3.69	0.58	2.26
10	1	27	3	11.99	11.99	11.99
11	2	22/22	1	2.03	0.57	1.46
12	4	24/24/24/22	4	6.53	0.52	2.64
13	5	22/22/22/22/22	1	1.22	0.17	0.32
14	3	22/22/22	1	2.09	0.36	1.28
15	1	22	1	0.67	0.67	0.67
16	1	27	3	14.68	14.68	14.68
17	6	30/25/25/30/25/25	5	21.49	0.20	17.13
18	1	30	5	29.07	29.07	29.07
19	2	22/18	1	4.55	1.26	3.28
20	3	20/20/20	1	1.38	0.19	0.72

**Table 2:** SRS vs. fSRS considerations SRS - stereotactic radiosurgery; fSRS - fractionated stereotactic radiosurgery; OAR - organ at risk volume; V_12 _- the volume of normal brain receiving 12 Gy

SRS	fSRS
Target size < 3.0 cm	Target size > 3.0 cm
Sufficient OAR separation	Adjacent OAR
Targets well distributed (low dose bridging)	Targets clustered; close proximity (increased dose bridging)
V_12_ ( <5-10cc)	V_12_ ( >10cc)

Margins ranged from 1.0 to 2.0 mm, as shown in Table 3. The margin used for each patient is a function of setup uncertainty, including residual setup error after Brainlab (Munich, Germany) ExacTrac® imaging corrections at each couch position. This is to account for the effects of residual rotational uncertainties, which can have a more profound impact on targets located at greater distances from the isocenter [16]. Additionally, Sagawa et al. found that rotational setup errors caused non-negligible underdosage of the PTV if uncorrected [17]. At our institution, the GTV to clinical target volume (CTV) margin is zero. For most targets within 3.0 cm of the isocenter, a 1.0-1.5 mm CTV to PTV margin is used, and targets beyond 3.0 cm typically receive a 1.5-2.0 mm CTV to PTV margin. Keeping residual setup error minimized with the utilization of ExacTrac at non-coplanar couch positions, according to Table 3 tolerances, allows for a smaller margin selection. This is particularly important because recent publications have demonstrated that margins on the order of 3.0 mm increase the potential risk of radionecrosis, and expanding the margin beyond 1.0 mm is not associated with improved local control [18-19]. Treatment of postoperative cavities with fSRS and a minimum of 2.0 mm margin is shown to be effective [20].

**Table 3:** CTV to PTV margin definitions CTV - clinical target volume; PTV - planning target volume (*) - used for single targets

Margin (mm)	Exactrac translational tolerance (mm)	Exactrac rotational tolerance (degrees)	Distance from Isocenter (mm)
1.0	0.8	0.8	0 (*)
1.0 - 1.5	0.5	0.5	0 - 30
1.5 - 2.0	0.5	0.5	30 - 70

MME v2.0 plan generation

For MME v2.0, a standard five arc template was used, which demonstrated equivalent or better coverage than the MME v1.5 plans used for treatment. As with v1.5 plans used for treatment, the strategy was to develop the “best” plan with the new planning tools provided. If an OAR failed a dose constraint, that OAR was identified in the optimizer to improve and reduce the dose. All patient plan OARs were evaluated against dose constraints in the literature [21-22].

Data collection and statistical analysis

For each plan, calculations were performed with MME v2.0 and compared to MME v1.5 using a 6 FFF beam. The plan resolution was 1.5mm slice thickness with a 0.68 mm pixel size. CI, GI, V_12_, and mean brain dose were reported by the software and recorded for each patient. The reported Nakamura CI was converted to a Paddick CI, which is more widely utilized, for the results portion of this publication.

(1)

$Nakamura CI = \frac{TV x PIV}{TV^{_{PIV}^{2}}}$

(2)

$Paddick CI = \frac{TV_{PIV}^{2}}{TV x PIV}$

(3)

$GI = \frac{PIV_{50}}{PIV_{100}}$

The V_12_, is computed by subtracting the target volume (PTV) from the whole, normal brain volume. The mean value of each parameter (mean ± SD) was calculated for every patient. A Wilcoxon signed-rank test was utilized to obtain *p*-values and evaluate differences between v1.5 and v2.0 as well as the Varian HD120 MLC.

## Results

For Paddick CI, as value increases to a maximum of 1.0, this indicates improved conformity. As shown in Table 4, the conformity index increased significantly for MME v2.0 with Agility MLC (0.79 ± 0.05) and MME v2.0 with Agility MLC and jaw tracking (0.80 ± 0.05) compared to MME v1.5 (0.74 ± 0.09). The Varian HD120 MLC Paddick CI (0.82 ± 0.05) was slightly better than the Elekta Agility with jaw tracking (0.80 ± 0.05) (p<0.01). There was no significant difference between MME v2.0 with Agility MLC (0.79 ± 0.05) and MME v2.0 with Agility MLC and jaw tracking (0.80 ± 0.05) (p=0.178).

**Table 4:** Conformity index MME - Brainlab Elements Multiple Brain Mets (§) MME v2.0 Agility jaw tracking versus MME v2.0 Varian HD120 (‡) MME v2.0 Agility versus MME v2.0 Agility with jaw tracking

Patient	MME v1.5 Agility	MME v2.0 Agility	MME v2.0 Agility Jaw Tracking	MME v2.0 Varian HD120
1	0.63	0.67	0.69	0.73
2	0.74	0.74	0.75	0.77
3	0.78	0.78	0.78	0.82
4	0.49	0.80	0.82	0.83
5	0.68	0.72	0.78	0.81
6	0.80	0.78	0.78	0.75
7	0.82	0.83	0.80	0.85
8	0.84	0.88	0.86	0.90
9	0.77	0.79	0.83	0.84
10	0.79	0.85	0.85	0.86
11	0.79	0.83	0.80	0.84
12	0.76	0.83	0.87	0.85
13	0.61	0.6	0.68	0.72
14	0.74	0.75	0.82	0.82
15	0.81	0.85	0.83	0.88
16	0.78	0.84	0.84	0.87
17	0.73	0.84	0.81	0.80
18	0.75	0.8	0.79	0.81
19	0.84	0.87	0.85	0.87
20	0.69	0.77	0.79	0.81
Mean	0.74	0.79	0.80	0.82
SD	0.09	0.05	0.05	0.05
MIN	0.49	0.6	0.68	0.72
MAX	0.84	0.88	0.87	0.90
p-value		< 0.01	< 0.01	< 0.01
p-value			< 0.01(§)	
p-value		0.178(‡)		

The gradient index decreased significantly for MME v2.0 with Agility MLC (3.76 ± 0.76) and MME v2.0 with Agility MLC and jaw tracking (3.36 ± 0.65) compared to MME v1.5 (4.26 ± 0.91) (p<0.01), shown in Table 5. There was a significant decrease for MME v2.0 with Agility MLC (3.76 ± 0.76) and MME v2.0 with Agility MLC and jaw tracking (3.36 ± 0.65) (p<0.01). The Varian HD120 MLC (3.15 ± 0.56) gradient index was slightly better than the Elekta Agility with jaw tracking (3.36 ± 0.65), although not significant (p=0.017).

**Table 5:** Gradient index MME - Brainlab Elements Multiple Brain Mets (§) MME v2.0 Agility jaw tracking versus MME v2.0 Varian HD120 (‡) MME v2.0 Agility versus MME v2.0 Agility with jaw tracking

Patient	MME v1.5 Agility	MME v2.0 Agility	MME v2.0 Agility Jaw Tracking	MME v2.0 Varian HD120
1	5.29	4.83	3.99	3.80
2	5.12	4.60	4.25	3.59
3	4.40	3.90	3.64	3.41
4	4.26	3.73	3.63	3.37
5	5.00	3.88	3.08	3.32
6	5.28	4.50	3.47	3.60
7	4.82	3.39	3.13	3.35
8	3.41	2.89	2.69	2.54
9	4.20	3.70	3.54	3.25
10	2.55	2.38	2.30	2.25
11	4.24	3.87	3.32	3.11
12	3.63	3.34	3.05	3.12
13	5.47	4.32	4.39	4.11
14	4.38	4.04	3.57	3.23
15	4.86	4.28	2.87	2.97
16	2.77	2.50	2.36	2.33
17	4.80	4.53	4.44	4.36
18	2.43	2.39	2.31	2.30
19	3.68	3.54	3.18	2.92
20	4.57	4.68	3.91	3.35
Mean	4.26	3.76	3.36	3.15
SD	0.91	0.76	0.65	0.56
MIN	2.43	2.38	2.30	2.25
MAX	5.47	4.83	4.44	4.36
p-value		< 0.01	< 0.01	< 0.01
p-value			0.017(§)	
p-value		< 0.01(‡)		

The V_12_ was reduced for MME v2.0 with Agility MLC (16.15 ± 15.97) and MME v2.0 with Agility MLC and jaw tracking (15.01 ± 15.47) compared to MME v1.5 (18.44 ± 17.31) (p<0.01), shown in Table 6. Additionally, MME v2.0 with Agility MLC (16.15 ± 15.97) versus MME v2.0 with Agility MLC and jaw tracking (15.01 ± 15.47) showed a significant decrease (p<0.01). The Varian HD120 MLC (13.72 ± 14.38) V12 was better than the Elekta Agility with jaw tracking (15.01 ± 15.47) (p<0.01).

**Table 6:** Volume of normal brain receiving 12Gy (V12) MME - Brainlab Elements Multiple Brain Mets (§) MME v2.0 Agility jaw tracking versus MME v2.0 Varian HD120 (‡) MME v2.0 Agility versus MME v2.0 Agility with jaw tracking

Patient	MME v1.5 Agility (cm^3^)	MME v2.0 Agility (cm^3^)	MME v2.0 Agility Jaw Tracking (cm^3^)	MME v2.0 Varian HD120 (cm^3^)
1	9.86	8.32	7.04	6.16
2	14.29	12.88	11.69	9.03
3	41.42	41.56	39.69	32.57
4	41.63	34.41	33.07	30.53
5	7.63	5.02	4.16	3.74
6	5.68	4.87	4.00	4.31
7	1.84	1.20	1.16	1.14
8	6.95	4.77	4.58	3.67
9	10.97	9.22	8.26	7.22
10	23.16	19.32	18.49	17.10
11	6.97	5.85	5.23	4.42
12	24.91	20.54	19.50	15.91
13	6.58	5.26	4.74	4.04
14	7.60	6.65	5.13	4.67
15	2.69	2.17	1.33	1.27
16	41.14	33.55	31.07	29.00
17	53.22	50.52	47.59	47.20
18	50.74	45.97	44.72	41.83
19	6.78	6.04	5.50	4.69
20	4.74	4.98	3.19	5.96
Mean	18.44	16.15	15.01	13.72
SD	17.31	15.97	15.47	14.38
MIN	1.84	1.20	1.16	1.14
MAX	53.22	50.52	47.59	47.20
p-value		< 0.01	< 0.01	< 0.01
p-value			< 0.01(§)	
p-value		< 0.01(‡)		

The brain mean dose was reduced for MME v2.0 with Agility MLC (185 ± 109) and MME v2.0 with Agility MLC and jaw tracking (187 ± 110) compared to MME v1.5 (193 ± 116), (p=0.02). Agility MLC and jaw tracking (187 ± 110) showed an increase compared to Agility MLC (185 ± 109) (p=0.92) (Table 7). The Varian HD120 MLC mean brain dose (169 ± 109) was substantially better than the Elekta Agility with jaw tracking (187 ± 110) (p<0.01).

**Table 7:** Brain mean dose MME - Brainlab Elements Multiple Brain Mets (§) MME v2.0 Agility jaw tracking versus MME v2.0 Varian HD120 (‡) MME v2.0 Agility versus MME v2.0 Agility with jaw tracking

Patient	MME v1.5 Agility (cGy)	MME v2.0 (cGy)	MME v2.0 Agility Jaw Tracking (cGy)	MME v2.0 Varian HD120 (cGy)
1	159	152	186	132
2	185	171	187	147
3	394	356	360	321
4	403	374	390	374
5	118	121	135	104
6	111	107	120	109
7	42	41	41	22
8	90	86	84	68
9	162	150	145	139
10	196	173	173	145
11	126	117	112	94
12	300	308	302	285
13	158	168	169	153
14	152	150	139	133
15	51	49	44	28
16	236	211	213	182
17	418	421	412	420
18	336	302	302	275
19	134	136	131	120
20	106	113	96	121
Mean	193	185	187	169
SD	116	109	110	109
MIN	42	41	41	22
MAX	418	421	412	420
p-value		0.02	0.06	< 0.01
p-value			< 0.01(§)	
p-value		0.92(‡)		

## Discussion

MME v2.0, as compared to v1.5, demonstrated improvement for all parameters except for mean brain dose for the Elekta with Agility MLC. This is most likely due to additional arcs added during v2.0 planning to improve other dosimetric parameters. Further planning and software experience may allow for the improvement of this parameter. For the Agility MLC with jaw tracking, the feature demonstrated dosimetric enhancements except for CI and mean brain dose. This may be due to the fact that jaw tracking only influences dose on peripheral targets during a given arc pass; thus, the improvement is limited to those targets. It is important to note that peripheral targets change depending on the arc pass and table position developed during optimization.

As part of this study, we compared the dosimetric plan quality when treating with an Elekta Agility MLC versus the Varian HD120 MLC, both using the latest v2.0 software. Historically, a smaller MLC leaf width has been associated with an improvement in dose conformity, and this benefit was associated with intensity-modulated techniques and demonstrating improvement when moving from a 5.0 mm to 2.5 mm MLC leaf width [23]. Conversely, other investigators have concluded that the improvement provided by smaller leaves can be overcome with larger leaves by the use of a more sophisticated delivery technique such as intensity-modulated radiotherapy (IMRT) or volumetric-modulated arc therapy (VMAT) [24]. Further, it has been suggested that software solutions provide comparable target conformity, regardless of MLC width [25]. The Varian HD120 offered improvements in the CI, V12, and mean brain dose, but not for GI. While these differences were statistically significant, the absolute differences (mean CI 0.80 vs. 0.82) are likely only clinically significant for a limited group of patients, potentially those with substantially smaller PTV volumes or an extreme number of targets.

A unique planning challenge for MBM treated with a single isocenter and a VMAT approach is controlling low dose ‘spill’ into normal brain outside of the intended targets. This is due to several phenomena, including increased MLC leakage and the ‘island blocking problem,’ as reported by Kang et al. [26]. The island blocking problem occurs when two or more targets lie in the direct path of MLC leaf travel. If the MLC leaves are opened to optimize multiple target doses simultaneously, then the space containing normal brain tissue in between the targets will also be irradiated unintentionally. In addition to island blocking, MLC leakage is a function of vendor design and becomes more profound for larger fields, such as those found in MBM treated with VMAT. Both Elekta and Varian provide MLC designs with inherently low leaf transmission resulting in a lower dose spill to the normal brain when blocked by the leaves and jaws. Though the Varian has a higher MLC leaf transmission, it demonstrated a smaller mean brain dose and V12. This is most likely related to the tighter conformity afforded by the 2.5 mm leaves for central targets.

Many of the island blocking challenges can be overcome in VMAT planning with optimization of treatment geometry and delivery to ensure that targets are not aligned with MLC leaf travel [27]. Consequently, VMAT SRS solutions require measurement of patient-specific fluence QA before delivery, and due to the high dose, small field nature of the individual targets, tighter gamma index criteria is recommended [28].

**Figure 1:**
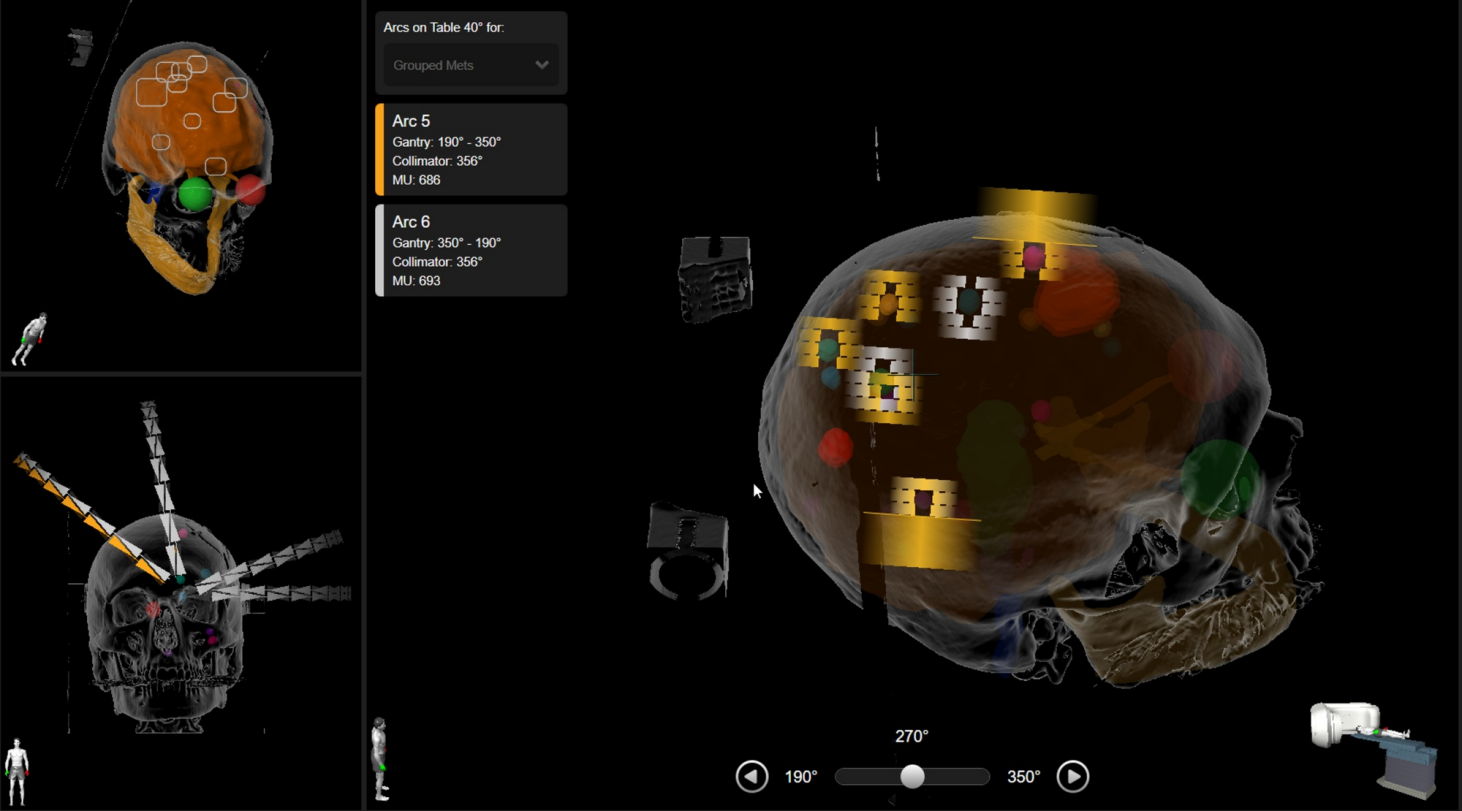
Treatment delivery showing multiple arc passes at a single couch angle The targets treated in the clockwise pass are shown with orange multileaf collimator outlines. The targets treated in the counterclockwise pass are shown with white multileaf collimator outlines.

Brainlab Elements overcomes the VMAT complexities of the island blocking problem by delivering multiple dynamic conformal arc (DCA) passes at a given couch angle. Primarily, when multiple targets lie in the direction of MLC travel, only select targets are treated during the first arc pass, and the MLC blocks select ones. On the next arc pass, the previously treated targets are blocked, and untreated targets are now treated. This ensures minimum normal tissue exposure outside of the intended targets, as shown in Figure 1. The details of the inverse optimized DCA algorithm and approach have been presented by Gevaert et al. [10].

**Figure 2:**
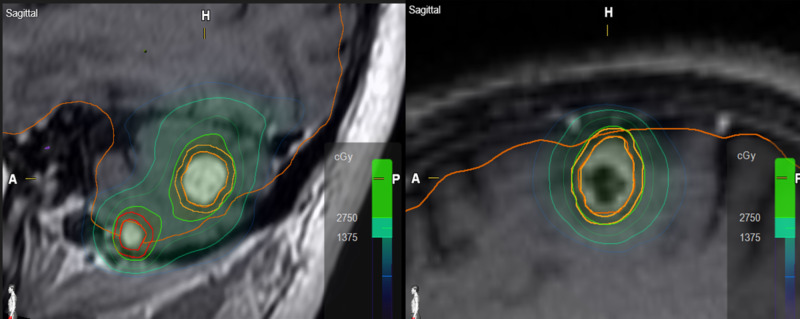
On the left side, a patient with multiple targets in close proximity demonstrating the mutually deteriorating effect on conformity and gradient indices. On the right, a single, isolated target demonstrating improved conformity and gradient indices.

An issue regarding target conformity and gradient index relates to the target volume and how the targets are spatially distributed with respect to each other. Targets close to each other will tend to have worse dose metrics as dose contributions to one target will have a mutually deteriorating effect on nearby targets when treated at the same time. Additionally, as the number of targets increases, or the total target volume increases, conformity and normal tissue sparing will decrease [29]. This was observed in cases involving clustered multiple targets (Figure 2). Given that calculating and interpreting CI and GI for clustered metastases is complicated and sometimes not even feasible, we have found that evaluating mean brain dose as a measure of plan quality particularly useful in those situations with clustered lesions or a large number of targets. The majority of these cases are treated with fSRS with a mean brain dose goal of 4-5 Gy and 3 Gy for five-fraction and three-fraction cases, respectively.

## Conclusions

Treatment of BM with Brainlab Elements Multiple Brain Mets SRS application v2.0 provides a robust, efficient, and cost-effective solution that requires less extensive department resources to plan, perform quality assurance, and deliver when compared to other available technologies. As demonstrated, using the Elekta Agility MLC with 5.0 mm leaves provides excellent target coverage and conformity for a range of SRS and fSRS cases. The software enhancements provided in MME v2.0 and the modest difference in Varian HD120 plans demonstrate further incremental benefits of improved software optimization and MLC design.
